# Supplementary material for: Static loading of the knee joint results in modified single leg landing biomechanics
Source: PLoS One. 2020 Feb 21;15(2):e0219648. doi: 10.1371/journal.pone.0219648 (PMC7034804; doi:10.1371/journal.pone.0219648)
Supplement: S1 Table — (DOCX) [file pone.0219648.s001.docx]

| MEANS |  |  |  |  |  |  |
| --- | --- | --- | --- | --- | --- | --- |
| Pre | time interval | |  |  |  |  |
| Muscle | 200-0 | 100-0 | 0-50 | 51-100 | 101-150 | 151-200 |
| BF | 0.152431 | 0.185972 | 0.189525 | 0.240963 | 0.179285 | 0.154235 |
| RF | 0.135055 | 0.192155 | 0.371267 | 0.551316 | 0.376579 | 0.213799 |
| SM | 0.165902 | 0.180679 | 0.314977 | 0.57631 | 0.409886 | 0.342265 |
| VL | 0.236692 | 0.228544 | 0.508688 | 0.773933 | 0.591581 | 0.381032 |
| VM | 0.093424 | 0.134069 | 0.251516 | 0.43683 | 0.301327 | 0.22344 |
|  |  |  |  |  |  |  |
| Post | time interval | |  |  |  |  |
| Muscle | 200-0 | 100-0 | 0-50 | 51-100 | 101-150 | 151-200 |
| BF | 0.086174 | 0.116771 | 0.125296 | 0.249862 | 0.185301 | 0.114733 |
| RF | 0.12764 | 0.173425 | 0.339452 | 0.531834 | 0.327685 | 0.20514 |
| SM | 0.154402 | 0.172514 | 0.350066 | 0.64478 | 0.523479 | 0.359796 |
| VL | 0.220309 | 0.295091 | 0.544354 | 0.728885 | 0.485178 | 0.365473 |
| VM | 0.082246 | 0.137613 | 0.274351 | 0.296819 | 0.184421 | 0.117094 |

| STANDARD DEVIATIONS | | |  |  |  |  |
| --- | --- | --- | --- | --- | --- | --- |
| Pre | time interval | |  |  |  |  |
| Muscle | 200-0 | 100-0 | 0-50 | 51-100 | 101-150 | 151-200 |
| BF | 0.288092 | 0.301044 | 0.267805 | 0.188456 | 0.110495 | 0.08058 |
| RF | 0.119548 | 0.179533 | 0.200103 | 0.316118 | 0.243227 | 0.117497 |
| SM | 0.192897 | 0.130096 | 0.29401 | 0.511673 | 0.28569 | 0.281162 |
| VL | 0.288496 | 0.138127 | 0.339993 | 0.48448 | 0.368107 | 0.322028 |
| VM | 0.149897 | 0.221822 | 0.397618 | 0.698164 | 0.446863 | 0.343603 |
|  |  |  |  |  |  |  |
| Post | time interval | |  |  |  |  |
| Muscle | 200-0 | 100-0 | 0-50 | 51-100 | 101-150 | 151-200 |
| BF | 0.055932 | 0.081677 | 0.052117 | 0.272646 | 0.135178 | 0.049991 |
| RF | 0.102085 | 0.144679 | 0.195394 | 0.319682 | 0.138663 | 0.073471 |
| SM | 0.184502 | 0.158394 | 0.328355 | 0.623303 | 0.551089 | 0.352665 |
| VL | 0.237708 | 0.304936 | 0.425752 | 0.473946 | 0.29874 | 0.274603 |
| VM | 0.114983 | 0.227776 | 0.483654 | 0.499231 | 0.280825 | 0.174267 |

**S1. Mean (sd) average EMG at pre- and postlanding at 200 and 100ms prior to landing, and at 50 ms intervals at landing.**
