# Supplementary material for: Static loading of the knee joint results in modified single leg landing biomechanics
Source: PLoS One. 2020 Feb 21;15(2):e0219648. doi: 10.1371/journal.pone.0219648 (PMC7034804; doi:10.1371/journal.pone.0219648)
Supplement: S2 Table — (DOCX) [file pone.0219648.s002.docx]

| condition | muscle | Mean | Std. Deviation | | condition | muscle | Mean | Std. Deviation |
| --- | --- | --- | --- | --- | --- | --- | --- | --- |
| pre | BF | 0.86506 | 0.561996 |  | post | BF | 0.973641 | 0.617574 |
|  | RF | 1.151254 | 0.620071 |  |  | RF | 1.175346 | 0.550462 |
|  | SM | 0.639673 | 0.305694 |  |  | SM | 0.809026 | 0.555181 |
|  | VL | 0.432708 | 0.448771 |  |  | VL | 0.452437 | 0.548723 |
|  | VM | 0.982636 | 0.615609 |  |  | VM | 1.042312 | 0.503083 |

**S2. Mean (sd) maximal EMG from each muscle group during pre- and post-landing conditions.**
