# Supplementary material for: Static loading of the knee joint results in modified single leg landing biomechanics
Source: PLoS One. 2020 Feb 21;15(2):e0219648. doi: 10.1371/journal.pone.0219648 (PMC7034804; doi:10.1371/journal.pone.0219648)
Supplement: S3 Table — (DOCX) [file pone.0219648.s003.docx]

| condition | interval | Mean | SD |  | condition | interval | Mean | SD |
| --- | --- | --- | --- | --- | --- | --- | --- | --- |
| pre | t0-50 | 977.493 | 313.8 |  | post | t0-50 | 903.749 | 328.9 |
|  | t50-100 | 1711.83 | 434.2 |  |  | t50-100 | 1610.39 | 540.3 |
|  | t100-150 | 1400.13 | 325.2 |  |  | t100-150 | 1310.46 | 440.8 |
|  | t150-200 | 1099.43 | 335.3 |  |  | t150-200 | 1100.03 | 366.4 |

**S3. Mean (sd) maximal VGRFs at each 50ms interval of pre- and postlanding up to 200 ms.**
