# Supplementary material for: Static loading of the knee joint results in modified single leg landing biomechanics
Source: PLoS One. 2020 Feb 21;15(2):e0219648. doi: 10.1371/journal.pone.0219648 (PMC7034804; doi:10.1371/journal.pone.0219648)
Supplement: Attachment — Submitted filename: Response to Reviwers.docx [file pone.0219648.s004.docx]

Dear Dr. Mirkov,

The suggestions and comments posed by you are the reviewers are greatly appreciated. After reviewing the feedback, the manuscript was revised to better communicate the intensions of the study. A manuscript with the track changes, to show the edits made, and a clean copy of the revised manuscript have been provided. Reponses to the reviewers have been indicated with R, and in bold type.

Thank you.

Response to Reviewers

Journal Requirements:

1. When submitting your revision, we need you to address these additional requirements.

Please ensure that your manuscript meets PLOS ONE's style requirements, including those for file naming. The PLOS ONE style templates can be found at
http://www.journals.plos.org/plosone/s/file?id=wjVg/PLOSOne_formatting_sample_main_body.pdf and <http://www.journals.plos.org/plosone/s/file?id=ba62/PLOSOne_formatting_sample_title_authors_affiliations.pdf>

**R: Thank you for this comment. The manuscript has been formatted to the specifications of the journal.**

2. Thank you for including your ethics statement: "Southern Illinois University Carbondale Human Subject Committee
Approval # 15277
written consent was provided by each participant"
a. Please amend your current ethics statement to confirm that your named institutional review board or ethics committee specifically approved this study.
b. Once you have amended this/these statement(s) in the Methods section of the manuscript, please add the same text to the “Ethics Statement” field of the submission form (via “Edit Submission”).
For additional information about PLOS ONE ethical requirements for human subjects research, please refer to http://journals.plos.org/plosone/s/submission-guidelines#loc-human-subjects-research

**R: The ethical statement has been amended and added as requested (p.5. lines 106-108).**

Reviewers' comments:

Reviewer #1: Research is conducted according the paper guidelines. Methodology and project design are set correctly. The only objection is the small number of subjects who participated in the study. It is not possible to make a quality conclusion based on such a number. It would be necessary to increase the number and adjust the statistical analysis in order to reach a good conclusion.

**R: Thank you for highlighting this item. It is believed that a quality conclusion can be established, based upon the reported data. The sample size was believed to be sufficient for this study. Areas of analysis have been streamlined to better present the information. As far as the statistical analysis is concerned, the explanation of the statistical procedures have been revised to more accurately describe how the data were assessed (p.11, lines 225-238).**

Reviewer #2: Review the manuscript titled “Static loading of the knee joint results in modified single leg landing biomechanics”
Manuscript Number: PONE-D-19-17819

The aim of this study was to investigate the muscular, kinetic and kinematic response of the lower limb during landing following anterior passive loading of the capsuloligamentous structures of the knee joint. The experimental setup used in the present study was similar to the one used by Chu et al (2003) who also investigated the effect of creep that developed in the anterior cruciate ligament due to prolonged static loading on the reflexive activation of the associated musculature.
Even though the scientific value of this study is unquestionable, as it enlightens the mechanisms that underlie knee injuries, there are some issues that require author’s respond, particularly with regard to the method, the statistical analysis of the research data as well as the presentation and discussion of the results, before the study is considered for publication.

General comments
Method
• The authors should justify the choice of loads that they implemented for static loading of the knee joint as these could be considered light to mild in comparison to the loads that are developed in sporting activities such as during landing. This issue should be discussed more thoroughly and possibly introduced as one of the limitations of the present study.

**R: This is a very good issue presented by the reviewer. The load type was performed to replicate the study performed by Chu et al. (2003). However, as the reviewer suggests, these loads may not have been sufficient for some participants. This has been added as a limitation of the study (p. 22 lines 452-456).**

• The authors should also justify their choice to use the dominant leg (the one the participants use for kicking a ball) in their study as previous studies have demonstrated significant differences between the dominant and non-dominant leg regarding knee joint stability during unilateral landing (Ludwig et al 2017; Herrington 2011).

**R: The use of the dominant leg has been used throughout the literature (Heebner et al., 2017; Jenkins et al., 2017; Kernozek et al., 2008). However, this does not discount the reviewer’s suggestion. This has been added as a limitation and framed as a topic for future work (p.22, lines 456-459).**

• Given the complexity of the experiment, it is suggested that the researchers provide photographic material for the experimental set up and procedure.

**R: Additional figure have been added to illustrate the experimental loading and landing protocols (p. 8 and 9).**

Statistical analysis
The general sense that someone gets from reading this manuscript is data over-analysis. The authors performed several statistical tests in order to identify potential effect of static loading of the knee’s passive structures during landing. However, there is a mismatch (in my opinion) between the statistical tests that were performed, the presentation of the Results and the data listed or depicted in Tables and Graphs, respectively. More specifically:
• The authors performed a Two-Way (condition x muscle) ANOVA to compare average and maximum normalized EMG of each muscle involved during landing between pre and post loading conditions (the results are listed in Table 1); they also performed a 5 (muscles) x 2 (conditions) x 4 (time intervals) Two-Way ANOVA for average normalized EMG (the results are graphically presented in Figure 1). It is clear that in the latter case the authors attempted to investigate how muscles’ EMG behave over time during landing; however in Figure 1 the normalized EMG that was recorded before landing (200-0 and 100-0 ms) is also depicted. Why the authors did not include the pre-landing EMG’s in the statistical analysis? Their decision should be is justified and, in any case, they should consider modifying Figure 1, as in its current state it seems that both pre-landing and during landing EMG responses were also compared.

**R: Thank you for finding these issues in the statistical analysis section. For clarity, the explanation of the data analysis for each dependent variable has been revised. When EMG analysis is conducted, the 200 ms before and 200 ms after landing are explicitly stated.**

• Similarly, EMG muscles ratios have been compare with two different statistical tests. A muscle ratio x condition Two-Way ANOVA was used to compare average and maximum EMG ratios between the pre- and post-loading condition (the results are listed in Table 3 and 4) and again a 6 (muscle ratios) x 2 (conditions) x 5 (time intervals) Two-Way ANOVA for muscles EMG ratios (results are graphically presented in Figure 2). In this case (i) the authors did not report whether muscles EMG ratios were based on the average or the maximum EMG activity, (ii) they included 5 time intervals whilst the time intervals during landing were 4; which was the 5th time interval? Did the authors include in their analysis a pre-landing time interval (200-0 or 100-0 ms)? And if they do so, why they did not the same for average and maximum EMG activity of the muscles under investigation? (iii) an additional column was added in Figure 2 (the one that represents total EMG ratio for each time interval) but it should be clarified whether or not it was included in the statistical analysis. The authors should consider omitting the “total EMG ratio column” particularly if its scientific value is meaningless as it confuses the reader (see next comment).

**R: To clarify the data and simplify the Results section, the ratio data have now been excluded from the data analysis.**

• In both aforementioned cases the “overall” average and maximum EMG as well as the “overall” muscle EMG ratios is presented in Tables 2-4. In Figure 2 and Figure 3 a “total” EMG ratio column and a “combined” vertical ground reaction forces column (VGRF), respectively is also presented. The authors should justify whether (i) the “overall”, “total” or “combined” are terms that were used interchangeably, (ii) these terms represent data obtained at the pre-landing and during landing condition (which in my opinion make sense) or data obtained before loading and after loading of the passive knee structures. The legends in Tables 3 and 4 add more confusion to the reader as it is not clear whether the data listed in Tables represent “… average and maximal muscle ratios during landing phase between pre and post landing conditions” or “… loading conditions”. In any case the authors should consider omitting the “overall”, “total” or “combined” data, as in my opinion this information neither adds more scientific value to this research nor it is thoroughly discussed.

**R: There is agreement with the reviewer that the “overall” and “total” data do not provide meaningful information. These have been removed from the figures and tables. The Figure legends and table legends have been updated to reflect a clearer reflection of the presented data.**

• For VGRF the authors (i) reported the test that they used in statistical analysis, (ii) in the Results section they reported no statistical differences between conditions with regard to absolute peak and normalized peak forces but (iii) in the Discussion section they stated that “significant reductions in the average VGRF after static loading of the knee was present” (Page 20, Lines 429-430). This information was probably extracted from the graph in Figure 3 (?), but the authors neglected to report the level of statistical significances either in the text or in the graph.

**R: These VGRF results have been included in the Results section of the revised manuscript (p. 17, lines 308-310). Additionally, the normalized forces have been omitted from the revised manuscript.**

• The statistical tests used for maximum and minimum angular displacement, angular velocities and ROM comparisons between conditions were not reported.

**R: These statistical tests are now included in the revised manuscript (p. 11, lines 225-227).**

Discussion

The authors should discuss the limitations of the study and propose future research based on the findings and / or the methodological Inadequacies of the present study.

**R: A section has been included to highlight the limitations and potential future studies related to these limitations (p. 22, lines 452-461).**

Minor comments
Page 3, Line 47: The authors should consider replacing “… stress and strains which …” with “… stress and strains, which…”

**R: This has been changed, as suggested by the reviewer.**

Page 3, Line 61: The authors should clarify whether they are referring to the anatomical (malalignment of mechanical axes) or dynamic “… knee valgus …”

**R: This point has been clarified to the dynamics of the movement.**

Page 4, Line 68: The authors should consider replacing “…musculotendonous…” with “…musculotendinous…”

**R: This spelling has been corrected throughout the document.**

Page 6, Line 126: The authors should describe the technique used, if any, for identification of motor points and/or cite related references.

**R: References for electrode placement have been provided in the methods (p. 6-7, lines 127-131).**

Page 7, Lines 148-152: The authors should clarify the number of test trials that were actually included in data analysis as according to their protocol 10 test trials were required but eventually “… 5 sufficient trials…” were enough. Does this mean that the average of 5 trials out of the 10 was included in the data analysis and, if yes, which trials were discarded if more than 5 trials were recorded?

**R: This has been clarified in the revised document.**

Page 13, Line 276: The authors should replace (or not) the “… pre and post landing conditions” with “… pre and post loading conditions” in the legend of Table 3. The same applies for the legend of Table 4 (Page 14).

**R: These changes have been made in the revised manuscript.**

Page 13, Table 3: The authors should consider omitting the “overall” data (unless they explain and justify its use) since, in my opinion, it is confusing, not related with the initial hypothesis and eventually not discussed, although it appears that is the only data sets that present statistical significances. The same applies for the legend of Table 4 (Page 14).

**R: As stated previously, this has been deleted from the figures, tables, and text of the manuscript.**

Page 15, Line 307: The authors should consider replacing “… (p>0.071)” with “… (p<0.071)”

**R: This has been changed.**

Page 19, Lines 400-401: The authors should consider replacing “… overall knee rotation at landing” with “… overall knee flexion at landing”. They should also consider modifying Table 5 as in this Table the minimum and maximum, but no “overall”, displacement is listed.

**R: These changes have been made to the manuscript.**

Page 19, Line 407: The authors should consider replacing “… (Table 4)” should with “… (Table 5)” as the latter one display data values of joints angular displacements while the former displays data values of EMG muscle ratios.

**R: This has been corrected in the manuscript.**

Page 19, Lines 410-411: The authors should specify the negative values in both the text and in Tables 5 and 6.

**R: This has been updated in the revised manuscript.**

Figure 1: The units for NEMG should be added in the vertical axis

**R: There are no units of normalized EMG. Rather, the data are provided with reference to the maximal EMG value of the respective muscle.**

Figure 1-3: The author should use the same title in the horizontal axes (Time or Time intervals) in all Figures. The levels of statistical significance may also be indicated in a revised version of the manuscript provided that the proposed amendments have been taken into account.
Figure 3: The legend should be modified according to the other graphs for consistency. For example: Pre-loading ν instead of Pre – black

**R: These changes have been made in the revised manuscript.**
