# Supplementary material for: Static loading of the knee joint results in modified single leg landing biomechanics
Source: PLoS One. 2020 Feb 21;15(2):e0219648. doi: 10.1371/journal.pone.0219648 (PMC7034804; doi:10.1371/journal.pone.0219648)
Supplement: Attachment — Submitted filename: Reviewers comments for PONE-D-19-17819.R1.docx [file pone.0219648.s005.docx]

**Review of the manuscript titled “Static loading of the knee joint results in modified single leg landing biomechanics”. Manuscript Number: PONE-D-19-17819.R1**

The investigator of this study responded to all the comments and queries that were highlighted after reviewing the first version of the manuscript. However, there are several points that should be clarified, particularly with regard to the statistical analysis of the research data as well as the presentation and discussion of the results, before the study is considered for publication.

**Abstract**

**Page 2, Lines 38-39:** The authors should clarify the abbreviations **aVGRF** and **NAEMG** as they do not appear in the text

**Methods**

**Pages 10-11, Lines 215-219:** The author should consider re-writing the sentence following the suggestions presented below.

**Statistical analysis**

**Page 11, Lines 224-234:** The author should **(i)** clarify why Pre- vs. Post- comparisons, for angular displacement and velocity variables as well as for average and maximal forces, RFD and VGRF, are performed with a One-Way Analysis Of Variance and not a paired-t test, **(ii)** report the statistical test that he used for the analysis of the Force Timing-related data, **(iii)** consider to refer to the statistical tests used for each variable in the same order that it is presented in the Results section and discussed in the Discussion section. Alternatively, the statistical tests could be presented in groups (e.g. first the statistical test used for paired comparisons and then the ANOVA tests).

**Results**

**Pages 12-16:** The author should specify what the “pre- and post- landing condition” and the “pre- and post-loading condition” are. It appears that (i) the terms “**pre-** and **post-** **landing”** are used when the comparisons are intended to assess the effect of the pre-landing-induced feed forward mechanism on certain variables (e.g. muscle activity) and (ii) the terms “**pre-** and **post-loading”** when the comparisons aimed to assess the effect of passive loading of the knee on a particular variable.

**An example:** Table 2 presents comparisons between pre- and post-landing conditions for “Overall Average and Maximum NEMG” data. This is okay since the author stated (Pages 10-11, Lines 215-219) that such comparisons will provide information on the effect of the feed forward mechanism. However, it is not clear whether this represents the data that recorded **before (pre-)** or **after (post-)** passive loading of the knee. The author should (i) consider presenting pre- and post-loading and pre- and post-landing data in Tables or Figures as he did for the average NEMG data (Figure 3) and (ii) perform Two-Way ANOVAs for the maximum NEMG, as well as for all the other variables, as he did for average NEMG and VGRF.

Such clarifications should be made in the following cases, but generally this should be done whenever is deemed necessary.

**Page 12, Line 250:** In the text

**Page 13, Line 253 and 255:** In the text and the legend of Table 2. It should be clarified whether the data presented refers to the pre-loading or the post-loading condition

**Page 13, Line 257:** Consider replacing **“…during drop landing conditions.”** with **“…during drop landing.”**

**Page 13, Line 263-272:** The author should **(i)** clarify in the text the "conditions" that he is referred to and **(ii)** report in the legend of Figure 3 that the data is referred to both pre- and post-loading conditions.

**Page 14, Line 275 and 279:** In the text and legend of Table 3 for angular displacement data

**Page 14, Line 282-286 and Page 15, Line 294:** In the text and legend of Table 4 for angular velocity data

**Page 15, Line 296-297 and 303-304:** In the text and legend of Table 5 for joint ROM data

**Page 15-16, Lines 306-310:** In the text and legend (**Page 16, Line 312-314)** of Figure 4 for VGRF (How is it possible to record VGRF before landing?).

**Page 16, Line 316:** The author should consider defining in the Method section the Force Timing variable and report the statistical test that it was used for its analysis before presenting the related Results, as this variable appears for the first time in this section. Furthermore, Figure 4 presents maximum VGRF and not Force Timing data the values of which remain unspecified.

**Page 12, Line 252:** The author should consider replacing **“Maximal”** with **“Maximum”** and **“EMG”** with **“NEMG”** wherever is deemed appropriate.

**Table 2 and Figure 3:** The presentation of the values in Table 2 and Figure 3 for Average NEMG is inconsistent. The values presented in Table 2 are percentages of MVIE whilst the values presented in Figure 3 vary between 0-1.5 but remain unspecified. The authors should consider revising either the data in the Table or the data in the Figure.

**Tables 3-5:** The author should clarify the movement of ankle - dorsiflexion or plantarflexion - that is presented in the Tables and to explain the negative numbers.

**Figure 4:** The author should consider replacing the labels **“Pre-landing”** and **“Post-landing”** with **“Pre-loading”** and **“Post-loading”** that is presented within Figure 4.

**Discussion**

The discussion is very well written but the author must explain which conditions were compared. This is particularly important for the understanding of the discussion related to the Kinematic and Kinetic data.
