# Supplementary material for: Static loading of the knee joint results in modified single leg landing biomechanics
Source: PLoS One. 2020 Feb 21;15(2):e0219648. doi: 10.1371/journal.pone.0219648 (PMC7034804; doi:10.1371/journal.pone.0219648)
Supplement: Attachment — Submitted filename: Response to Reviewers 2 revision.docx [file pone.0219648.s006.docx]

Dear Dr. Mirkov,

I want to thank you and the reviewers for providing further insight into the submitted manuscript titled, *Static loading of the knee joint results in modified single leg landing biomechanics.* The manuscript has been revised based upon the reviewers’ suggestions. Below are the responses to the specific comments posed by the reviewers. A marked up version and a clean version of the manuscript have been submitted. I look forward to hearing from you.

Sincerely,

Michael W. Olson, PhD

**Review of the manuscript titled “Static loading of the knee joint results in modified single leg landing biomechanics”. Manuscript Number: PONE-D-19-17819.R1**

The investigator of this study responded to all the comments and queries that were highlighted after reviewing the first version of the manuscript. However, there are several points that should be clarified, particularly with regard to the statistical analysis of the research data as well as the presentation and discussion of the results, before the study is considered for publication.

**Abstract**

**Page 2, Lines 38-39:** The authors should clarify the abbreviations **aVGRF** and **NAEMG** as they do not appear in the text

**R: The abbreviations have been modified to agree with the abbreviations in the text.**

**Methods**

**Pages 10-11, Lines 215-219:** The author should consider re-writing the sentence following the suggestions presented below.

**R: please see the response after the following reviewer suggestion**

**Statistical analysis**

**Page 11, Lines 224-234:** The author should **(i)** clarify why Pre- vs. Post- comparisons, for angular displacement and velocity variables as well as for average and maximal forces, RFD and VGRF, are performed with a One-Way Analysis Of Variance and not a paired-t test, **(ii)** report the statistical test that he used for the analysis of the Force Timing-related data, **(iii)** consider to refer to the statistical tests used for each variable in the same order that it is presented in the Results section and discussed in the Discussion section. Alternatively, the statistical tests could be presented in groups (e.g. first the statistical test used for paired comparisons and then the ANOVA tests).

**R: It was believed that the ANOVA test was a valid statistical assessment of the values provided between the two conditions. In viewing the one-way ANOVA and paired-t-test options, both would provide the same outcome, thus the one-way ANOVA has been reported.**

**Results**

**Pages 12-16:** The author should specify what the “pre- and post- landing condition” and the “pre- and post-loading condition” are. It appears that (i) the terms “**pre-** and **post-** **landing”** are used when the comparisons are intended to assess the effect of the pre-landing-induced feed forward mechanism on certain variables (e.g. muscle activity) and (ii) the terms “**pre-** and **post-loading”** when the comparisons aimed to assess the effect of passive loading of the knee on a particular variable.

**R: The text has been edited to clarify pre-loading landing and post-loading landing conditions – these are the only “conditions”, as defined in the Methods section**

**An example:** Table 2 presents comparisons between pre- and post-landing conditions for “Overall Average and Maximum NEMG” data. This is okay since the author stated (Pages 10-11, Lines 215-219) that such comparisons will provide information on the effect of the feed forward mechanism. However, it is not clear whether this represents the data that recorded **before (pre-)** or **after (post-)** passive loading of the knee. The author should (i) consider presenting pre- and post-loading and pre- and post-landing data in Tables or Figures as he did for the average NEMG data (Figure 3) and (ii) perform Two-Way ANOVAs for the maximum NEMG, as well as for all the other variables, as he did for average NEMG and VGRF.

Such clarifications should be made in the following cases, but generally this should be done whenever is deemed necessary.

**R: All comparisons are between pre-loading landing and post-loading landing conditions. This nomenclature has been modified to reflect the clarity of the conditions in the text. The NEMG was evaluated 200 ms pre-landing and 200 ms post-landing, while the VGRF data were evaluated 200 ms post-landing in each condition. The data are presented in either figures or tables in the manuscript. The “overall average and maximal” values were removed for the first revision, so it is unclear what the reviewer is viewing.**

**Page 12, Line 250:** In the text

**R: the conditions have been defined in the Methods section for clarity**

**Page 13, Line 253 and 255:** In the text and the legend of Table 2. It should be clarified whether the data presented refers to the pre-loading or the post-loading condition

**R: this has been clarified**

**Page 13, Line 257:** Consider replacing **“…during drop landing conditions.”** with **“…during drop landing.”**

**R: this has been clarified**

**Page 13, Line 263-272:** The author should **(i)** clarify in the text the "conditions" that he is referred to and **(ii)** report in the legend of Figure 3 that the data is referred to both pre- and post-loading conditions.

**R: these have been clarified**

**Page 14, Line 275 and 279:** In the text and legend of Table 3 for angular displacement data

**R: this has been clarified**

**Page 14, Line 282-286 and Page 15, Line 294:** In the text and legend of Table 4 for angular velocity data

**R: this has been clarified**

**Page 15, Line 296-297 and 303-304:** In the text and legend of Table 5 for joint ROM data

**R: this has been clarified**

**Page 15-16, Lines 306-310:** In the text and legend (**Page 16, Line 312-314)** of Figure 4 for VGRF (How is it possible to record VGRF before landing?).

**R: it is not. It is not clear what the reviewer is reading, but there has been no indication in this manuscript that forces were recorded before landing.**

**Page 16, Line 316:** The author should consider defining in the Method section the Force Timing variable and report the statistical test that it was used for its analysis before presenting the related Results, as this variable appears for the first time in this section. Furthermore, Figure 4 presents maximum VGRF and not Force Timing data the values of which remain unspecified.

**R: this has been defined in the Methods section: “A 2-way ANOVA (condition x time interval) was used to assess average VGRF data during the landing phase, while one-way ANOVAs were used to compare maximal VGRF values between conditions”, p.11, lines 231-234. The caption of the section has been changed to clarify this variable assessment.**

**Page 12, Line 252:** The author should consider replacing **“Maximal”** with **“Maximum”** and **“EMG”** with **“NEMG”** wherever is deemed appropriate.

**R: this has been clarified**

**Table 2 and Figure 3:** The presentation of the values in Table 2 and Figure 3 for Average NEMG is inconsistent. The values presented in Table 2 are percentages of MVIE whilst the values presented in Figure 3 vary between 0-1.5 but remain unspecified. The authors should consider revising either the data in the Table or the data in the Figure.

**R: this has been clarified in Table 2**

**Tables 3-5:** The author should clarify the movement of ankle - dorsiflexion or plantarflexion - that is presented in the Tables and to explain the negative numbers.

**R: this has been clarified in the footnote for tables 3. Table 4 provides angular velocity data and the signs are indicative of positive and negative rates of movement, respectively. Table 5 represents ROM and does not require and explanation for the ankle**

**Figure 4:** The author should consider replacing the labels **“Pre-landing”** and **“Post-landing”** with **“Pre-loading”** and **“Post-loading”** that is presented within Figure 4.

**R: this has been modified.**

**Discussion**

The discussion is very well written but the author must explain which conditions were compared. This is particularly important for the understanding of the discussion related to the Kinematic and Kinetic data.

**R: the conditions have been claried in the Discussion section**
